# Distribution Approach to Local Volatility for European Options in the Merton Model with Stochastic Interest Rates

**DOI:** 10.3390/e27030320

**Published:** 2025-03-19

**Authors:** Piotr Nowak, Dariusz Gatarek

**Affiliations:** Systems Research Institute, Polish Academy of Sciences, Newelska 6, 01-447 Warsaw, Poland; gatarek@ibspan.waw.pl

**Keywords:** Dupire formula, European option, Merton model, local volatility, Schwartz distributions

## Abstract

The Dupire formula is a very useful tool for pricing financial derivatives. This paper is dedicated to deriving the aforementioned formula for the European call option in the space of distributions by applying a mathematically rigorous approach developed in our previous paper concerning the case of the Margrabe option. We assume that the underlying asset is described by the Merton jump-diffusion model. Using this stochastic process allows us to take into account jumps in the price of the considered asset. Moreover, we assume that the instantaneous interest rate follows the Merton model (1973). Therefore, in contrast to the models combining a constant interest rate and a continuous underlying asset price process, frequently observed in the literature, applying both stochastic processes could accurately reflect financial market behaviour. Moreover, we illustrate the possibility of using the minimal entropy martingale measure as the risk-neutral measure in our approach.

## 1. Introduction

More than thirty years ago, it was observed that the commonly used Black–Scholes model does not fully describe the financial reality. One of the phenomena confirming this, known as the implied volatility smile, is the dependence of option-implied volatility on option strike (see, e.g., [1]). Taking this phenomenon into account, Dupire [2] and Derman and Kani [3] introduced a deterministic function of the spot price of the underlying asset S and time to the Black–Scholes model for the description of the instantaneous volatility of S. The proposed form of instantaneous volatility initialised the class of local volatility models, preserving the advantages of the Black and Scholes approach.

The classical Dupire formula (or Dupire’s forward equation) for the no-arbitrage price c of the European call option with maturity t and strike price k at time zero takes the following form:

(1) $$\begin{array}{c} \frac{\partial c}{\partial t}=\frac{1}{2}{\overset{=}{\sigma }}^{2}\left(t,k\right)k^{2}\frac{\partial^{2}c}{\partial k^{2}}-r_{t}k\frac{\partial c}{\partial k} \end{array}$$

with

$$c=c\left(t,k\right)=\exp\left(-\int_{0}^{t}r_{u}du\right)E^{Q}\left[{\left(S_{t}-k\right)}_{+}\right]$$

for a deterministic instantaneous risk-free interest rate r; a traded stock with price S of the following form with respect to the risk-neutral measure Q:

$$S_{t}=S_{0}+\int_{0}^{t}r_{u}S_{u}du+\int_{0}^{t}\overset{=}{\sigma }\left(u,S_{u}\right)S_{u}dW_{u};$$

a deterministic local volatility function $\overset{=}{\sigma }$; and the standard Brownian motion W with respect to Q (see, e.g., [4]). The symbol $E^{Q}$ used above denotes the expected value with respect to Q.

Equation (1) enables the recovery of the volatility function from market prices of options (see, e.g., [5,6]).

One of the first extensions of Equation (1) was made by Andersen and Andreasen [7] for Markov jump-diffusions and by Carr et al. [8] for local Lévy models. The Dupire formula for the European call option was significantly extended by Bentata and Cont [9], who assumed a semimartingale model of the underlying asset and a deterministic bounded form of the instantaneous interest rate. Recent extensions of Dupire’s equation for the Margrabe options (see [10] and also [11] and references therein) were made by Gatarek and Jabłecki [12] and Nowak and Gatarek [13,14]. Moreover, Hambly et al. [15] considered the case of barrier options. In turn, Hainaut and Leonenko [16] derived the fractional version of the Dupire formula for invertible Lévy subordinators.

Most extensions of Equation (1) concern the case of a deterministic or stochastic form of the instantaneous interest rate while assuming that the underlying asset price process is continuous (see, e.g., [17,18,19] for the stochastic interest rate case). However, Benhamou et al. [20] considered a hybrid model consisting of a stochastic form of the interest rate and a discontinuous process describing the asset price.

The method of option pricing with the application of the Dupire formula has been continuously developing since the 1990s. Initially, this formula was derived and applied to European vanilla options within the Black–Scholes model. Subsequently, it has been used for other types of options and models of the underlying asset price. In many cases, however, the formula was derived in a heuristic manner, disregarding the verification of mathematical assumptions. This fact may result in doubts about the correctness and applicability of the aforementioned formula to various specific cases. For financial practitioners, these doubts have led the need to apply the strictly mathematically justified Dupire formula. This article and previous papers [13,14] address this need and expectation. Mathematical problems related to the non-differentiability of the pay-off function have led to the necessity of involving distributions. The importance of the mathematical correctness of the Dupire formula applied in financial practice was also highlighted by Bentata and Cont [9], who also used a distributional approach.

This paper aims to provide a mathematically rigorous proof of the Dupire formula for the European call option in the space of distributions. This proof is based on a generalised Dupire equation for the Margrabe option, proved in detail in [14] for processes of asset prices in the form of Levý-type stochastic integrals, satisfying the appropriate mathematical assumptions. In our approach, we assume that the underlying financial instrument is described by the model proposed by Merton in 1976 (see, e.g., [7]), containing a jump part, therefore allowing for jumps in this instrument price to be taken into account. In turn, the interest rate dynamics are in the form of an arithmetic Brownian motion, proposed in the Merton interest rate model of 1973 (see, e.g., [21]). Therefore, in contrast to continuous underlying asset price models with deterministic interest rates, frequently observed in the literature, both stochastic processes could accurately reflect financial market behaviour. The Dupire formula, covering the case considered in this paper, was derived in a simplified way, without the formal application of distributions, by Benhamou et al. [20]. The mentioned formula, leaving aside the question of distributions, is mathematically equivalent to the one we obtained in this paper. Our approach, based on a theorem strictly proved in [14], includes the verification of all the necessary assumptions and uses advanced mathematical finance methods and stochastic analysis techniques to obtain the Dupire formula for the European call option.

Moreover, we present the possibility of applying the minimal entropy martingale measure as the risk-neutral measure in the case of a jump-diffusion model for the underlying asset price and a constant instantaneous interest rate to obtain the Dupire formula based on the market parameters with respect to the physical probability measure P. The resulting Dupire formula is a slight generalisation (without assuming the form of the distribution of the jumps in the price of an underlying instrument) of its counterpart derived in [14], which was based on the parameters with respect to the risk-neutral measure Q. This formula was also obtained in other settings, in [7,9]. However, in this paper, we additionally show how the corresponding parameters with respect to measure P can be used in the resulting Dupire formula under the assumption that the risk-neutral probability measure Q is the minimal entropy martingale measure.

The Dupire formulas derived in this paper are based on the approach proposed in [14], where distribution-valued stochastic processes were used as a consequence of technical difficulties in applying the classical analytical methods for the non-differentiable option’s pay-off function. In particular, in the mentioned paper, to prove the generalised Dupire formula applied in this paper, a version of the Tanaka formula in the space of distributions was proved and used.

This paper is organised as follows. Section 2 presents the preliminaries concerning a discussion on applying differential equations to option pricing, notions used in the following parts of the paper concerning distributions, and stochastic integration with respect to a compensated random measure, as well as the theorems used in the following sections, including the generalised Dupire equation for the Margrabe option. In Section 3, we derive and prove the Dupire formula for the European call option under the assumptions of the stochastic models of the underlying asset and instantaneous interest rate. Section 4 is devoted to the application of the minimal entropy martingale measure to the case of the Dupire formula for the European option, where a jump-diffusion model of the underlying asset price and a constant instantaneous interest rate are assumed. In Section 5, we present a numerical example of applying the Dupire formula in [20], for a case similar to the one considered in this paper. Finally, Section 6 contains some concluding remarks.

## 2. Preliminaries

This section contains the appropriate notations, definitions, and theorems concerning the theories of distributions and stochastic integration, as well as the generalised Dupire formula, which was proved in [14] and used in our main theorem. We begin with a discussion on the application of differential equations to option pricing.

### 2.1. Application of Differential Equations to Option Pricing

Apart from a strictly probabilistic approach, including the application of methods of stochastic analysis, partial differential Equations (PDEs) play a key role in the valuation of derivative financial instruments. The first essential papers in this area were written by Black and Scholes [22] and Merton [23]. Since the publication of these papers, the theory of valuing options and other derivatives has been developed, and generalisations of previously used models have been introduced. Solutions of backward PDEs, with time and the underlying asset’s price as variables, were used to obtain option prices. For continuous models of the underlying assets’ prices, the application of backward PDEs for valuing various types of options, including path-dependent ones, was discussed, e.g., in [21,24,25].

In the case of the underlying asset’s price model with jumps, the counterpart of a PDE is a partial integro-differential Equation (PIDE) (see, e.g., [7,26]).

The pricing method mentioned above has also been recently generalised and applied to the case of defaultable derivatives, with pay-offs at random (stopping) times (see [27,28]).

A backward pricing equation enables us to obtain the price of a financial derivative for a given maturity and striking price. In turn, by solving the Dupire Equation (1) (or its generalisation), which is a forward equation, one obtains the prices of an option for various striking prices and maturities in the considered case of the underlying asset (see, e.g., [9]). The local volatility approach initially enabled the exact calibration of the Black–Scholes model to the volatility surface (see, e.g., [29]). The Dupire equation has also been successfully applied to other types of options and is a useful tool in financial practice.

### 2.2. Basic Notations

Let R, $B\left(R\right)$, $R_{+}$, and N be the set of real numbers, the σ-algebra of Borel subsets of R, the set of non-negative real numbers, and the set of positive integers, respectively.

The indicator function of a set *A* will be denoted by $1_{A}$.

For a non-empty open set $\Upsilon \subset R^{2}$, we denote by $L_{loc}^{1}\left(\Upsilon \right)$ the space of locally integrable functions on Υ; by $D\left(\Upsilon \right)$ the space of $C^{\infty }$-functions of compact support in Υ, being the test function space of general (also called Schwartz) distributions; and by $D^{\prime }\left(\Upsilon \right)$ the space of general distributions, i.e., the space of all continuous linear functionals on $D\left(\Upsilon \right)$. For details concerning the topologies on $D\left(\Upsilon \right)$ and $D^{\prime }\left(\Upsilon \right)$, we refer the reader to [30].

If $\Upsilon_{1}\subset \Upsilon_{2}\subset R^{2}$, both sets are open, $\Upsilon_{1}$ is non-empty, and $\iota_{\Upsilon_{2},\Upsilon_{1}}:D\left(\Upsilon_{1}\right)\to D\left(\Upsilon_{2}\right)$ is a continuous linear function which assigns to an element of $D\left(\Upsilon_{1}\right)$ its continuation by 0 to $\Upsilon_{2}$, then, we introduce a function $\iota_{\Upsilon_{1},\Upsilon_{2}}:D^{\prime }\left(\Upsilon_{2}\right)\to D^{\prime }\left(\Upsilon_{1}\right)$ given by

$$\iota_{\Upsilon_{1},\Upsilon_{2}}\left(\varphi \right)=\varphi \circ \iota_{\Upsilon_{2},\Upsilon_{1}},\;\;\varphi \in D^{\prime }\left(\Upsilon_{2}\right)$$

and the following notation

$$\begin{array}{cc} \; & D=\iota_{R^{2},\left(0,T\right)\times R}\left(D\left(\left(0,T\right)\times R\right)\right),\;D^{\prime }=\iota_{\left(0,T\right)\times R,R^{2}}\left(D^{\prime }\left(R^{2}\right)\right). \end{array}$$

We also introduce the following functions:

$$\begin{array}{cc} \; & g_{a}:R\to R,\;k\mapsto g_{a}\left(k\right)=1_{\left(0,\infty \right)}\left(a-k\right),\\ \; & j\left(s,h\right):R\to R,\;k\mapsto j\left(s,h\right)\left(k\right)={\left(s+h-k\right)}_{+}-{\left(s-k\right)}_{+}-hg_{s}\left(k\right) \end{array}$$

for a,s,h∈R.

Finally, we recall the definition of the notion of a finite transition kernel.

**Definition** **1.** *For measurable spaces $\left(\Omega_{1},A_{1}\right)$ and $\left(\Omega_{2},A_{2}\right)$, a mapping $\kappa :\Omega_{1}\times A_{2}\to \left[0,\infty \right)$ is said to be a finite transition kernel (from $\left(\Omega_{1},A_{1}\right)$ to $\left(\Omega_{2},A_{2}\right)$), if the following conditions hold:*

*(i)*  *$\omega_{1}\mapsto \kappa \left(\omega_{1},A_{2}\right)$ is $A_{1}$-measurable for an arbitrary $A_{2}\in A_{2}$.*
*(ii)*  *$A_{2}\mapsto \kappa \left(\omega_{1},A_{2}\right)$ is a finite measure on $\left(\Omega_{2},A_{2}\right)$ for an arbitrary $\omega_{1}\in \Omega_{1}$.*

### 2.3. Lévy-Itô Stochastic Integrals

The theory of real stochastic processes is presented, e.g., by Protter [31].

Let $\left(\Omega ,F,{\left(F_{t}\right)}_{t\in T},P\right)$ be a filtered probability space fulfilling the usual conditions, $T=\left[0,T\right]$ and T<∞. Let E be the expected value with respect to P.

We will use Lévy-type stochastic integrals as models of the considered underlying assets. Therefore, we present some elements of the theory concerning these stochastic processes.

We use definitions from [32] to introduce the notion of a Lévy process and the associated measures. Let a stochastic process $X={\left(X_{t}\right)}_{t\in T}$ taking values in R be a Lévy process, i.e., it is a cádlág, stochastically continuous, $F_{t}$-adapted process which has stationary, independent increments, and $X_{0}=0$ a.s.

We use the symbol N(dtdx) to denote the measure on B(T×(R∖{0})) given by

$$\begin{array}{cc} N(\left(t_{1},t_{2}\right]\times A)= & \;\mathrm{the}\;\mathrm{number}\;\mathrm{of}\;\mathrm{jumps}\;\mathrm{of}\;X\;\mathrm{in}\;\mathrm{the}\;\mathrm{time}\;\mathrm{interval}\;(t_{1},t_{2}]\\ \; & \mathrm{such}\;\mathrm{that}\;\Delta X_{s}\in A,\;s\in \left(t_{1},t_{2}\right], \end{array}$$

for $0\leq t_{1}<t_{2}\leq T$, A∈B(R∖{0}), called *the jump measure of* X (or *the Poisson random measure associated with* X), where B(T×(R∖{0})) and B(R∖{0}) is the σ-algebra of Borel subsets of T×(R∖{0}) and R∖{0}, respectively.

The following measure $\bar{\nu }$ on B(R∖{0})

$$\begin{array}{c} \bar{\nu }\left(A\right):=EN\left(\left(0,1\right]\times A\right) \end{array}$$

for A∈B(R∖{0}) is called *the Lévy measure of* X. The *compensated jump measure of* X (or the *compensated Poisson random measure associated with* X) is defined by

$$\begin{array}{c} q\left(dt\;dx\right):=N\left(dt\;dx\right)-\bar{\nu }\left(dx\right)\;dt. \end{array}$$

Furthermore, we assume that measure $\bar{\nu }$ is finite.

We present some definitions and facts concerning the theory of stochastic integration with respect to a compensated Poisson random measure of real functions discussed in [33], adapting them to the case of the finite time horizon T.

The symbol $\hat{P}$ will be used to denote the σ-algebra of subsets of T×Ω×(R∖{0}), generated by mappings g:T×Ω×(R∖{0})→R fulfilling the following conditions:

(a) For an arbitrary t∈T, the mapping Ω×(R∖{0})∋(ω,z)↦g(t,ω,z)∈R is $B\left(R\setminus \left\{0\right\}\right)⊗F_{t}/B\left(R\right)$-measurable.
(b) For an arbitrary (ω,z)∈Ω×(R∖{0}), the mapping T∋t↦g(t,ω,z)∈R is left-continuous.

A process g:T×Ω×(R∖{0})→R is said to be predictable if it is $\hat{P}/B\left(R\right)$-measurable (see [33] (Def. 3.2.1)).

Let $M_{loc}^{2}\left(\hat{P},\bar{\nu },R\right)$ be the space of predictable processes V:T×Ω×(R∖{0})→R satisfying, for each T∈(0,T], the inequality

$$E\int_{0}^{T}\int_{R\setminus \{0\}}{\left|V\left(t,\omega ,z\right)\right|}^{2}\bar{\nu }\left(dz\right)dt<\infty .$$

Then, [33] (Thm. 3.2.27) implies that for each T∈(0,T], there exists a stochastic integral of *V* with respect to *q*, with the notation $\int_{0}^{T}\int_{R\setminus \{0\}}V\left(t,\omega ,z\right)q\left(dt\;dz\right)$, as well as $\int_{0}^{T}\int_{R\setminus \{0\}}V\left(t,z\right)q\left(dt\;dz\right)$ or $\int_{0}^{T}\int_{R}V\left(t,z\right)q\left(dt\;dz\right)$ (in the shortened form), being a square-integrable random variable.

We assume T=T without loss of generality. For $V\in M_{loc}^{2}\left(\hat{P},\bar{\nu },R\right)$, 0≤a≤b≤T, and B∈B(R∖{0}), process $1_{\left(a,b\right]}1_{B}V\in M_{loc}^{2}\left(\hat{P},\bar{\nu },R\right)$, and we use the notation

$$\int_{a}^{b}\int_{B}V\left(t,z\right)q\left(dt\;dz\right):=\int_{0}^{T}\int_{R\setminus \{0\}}1_{\left(a,b\right]}1_{B}V\left(t,z\right)q\left(dt\;dz\right).$$

It was proved in [33] (see Thm. 3.3.2) that for all t∈T, $V_{1},V_{2}\in M_{loc}^{2}\left(\hat{P},\bar{\nu },R\right)$ and α,β∈R,

(i) $\int_{0}^{t}\int_{R\setminus \{0\}}\left(\alpha V_{1}\left(t,z\right)+\beta V_{2}\left(t,z\right)\right)q\left(dt\;dz\right)\;=\alpha \int_{0}^{t}\int_{R\setminus \{0\}}V_{1}\left(t,z\right)q\left(dt\;dz\right)+\beta \int_{0}^{t}\int_{R\setminus \{0\}}V_{2}\left(t,z\right)q\left(dt\;dz\right);$
(ii) (2) $$E\int_{0}^{t}\int_{R\setminus \{0\}}V_{1}\left(t,z\right)q\left(dt\;dz\right)=0;$$
(iii) $E|\int_{0}^{t}\int_{R\setminus \{0\}}V_{1}{\left(t,z\right)q\left(dt\;dz\right)|}^{2}=E\int_{0}^{t}\int_{R\setminus \{0\}}{\left|V_{1}\left(t,z\right)\right|}^{2}\bar{\nu }\left(dz\right)dt$;
(iv) ${\left(\int_{0}^{t}\int_{R\setminus \{0\}}V_{1}\left(t,z\right)q\left(dt\;dz\right)\right)}_{t\in T}$ has a cádlág modification, being a square-integrable martingale.

In the following part of the paper, we will treat the integrals of $V\in M_{loc}^{2}\left(\hat{P},\bar{\nu },R\right)$ with respect to *q* as cádlág processes.

Moreover, under the assumption $\bar{\nu }\left(D\right)<\infty$ for D∈B(R∖{0}) (which is always satisfied in this paper), by [33] (Proposition 3.4.5), it is possible to consider the integral of $V\in M_{loc}^{2}\left(\hat{P},\bar{\nu },R\right)$ with respect to N and for an arbitrary t∈T

(3) $$\begin{array}{c} \begin{array}{cc} \int_{0}^{t}\int_{D} & V\left(s,z\right)N\left(ds\;dz\right):=\sum_{s\in \left(0,t\right]:|\Delta X_{s}|>0}V\left(s,\Delta X_{s}\right)1_{D}\left(\Delta X_{s}\right)\\ \; & =\int_{0}^{t}\int_{D}V\left(s,z\right)q\left(ds\;dz\right)+\int_{0}^{t}\int_{D}V\left(s,z\right)\bar{\nu }\left(dz\right)ds. \end{array} \end{array}$$

A real semimartingale $X={\left(X\right)}_{t\in T}$ is called a Lévy-type stochastic integral (a particular case of a Lévy-type stochastic integral defined in [34]), if it is described by the formula

(4) $$\begin{array}{cc} dX_{t} & =G_{s}ds+\sum_{j=1}^{m}F_{s}^{j}dW_{s}^{j}+\int_{|x|<1}H\left(t,x\right)q\left(dt\;dx\right)+\int_{|x|\geq 1}K\left(t,x\right)N\left(dt\;dx\right), \end{array}$$

for $G,F^{j}\in M\left(T\right)$, 1≤j≤m, $H1_{\left\{|x|<1\right\}}\left(x\right)$, $K1_{\left\{|x|\geq 1\right\}}\left(x\right)\in M_{loc}^{2}\left(\hat{P},\bar{\nu },R\right)$, and the *m*-dimensional Brownian motion $B=(W^{1},W^{2},\ldots ,W^{m})$, independent of N, where M(T) is the space of all predictable processes U:T×Ω→R satisfying the inequality

$$E\int_{0}^{T}{\left|U\left(t\right)\right|}^{2}dt<\infty .$$

We will use the following version of Itô’s lemma from [34] (Thm. 4.4.7) in a one-dimensional case with the finite time horizon T.

**Theorem** **1.** *Let $X={\left(X\right)}_{t\in T}$ be a Lévy-type stochastic integral given by (4). Then, for each $f\in C^{2}\left(R\right)$, t∈T, a.s.*

(5) $$\begin{array}{c} \begin{array}{cc} f(X_{t}) & -f\left(X_{0}\right)=\int_{0}^{t}f^{\prime }\left(X_{s-}\right)dX_{s}^{c}+\frac{1}{2}\int_{0}^{t}f^{\prime \prime }\left(X_{s-}\right)d\left[X_{c}\right]\\ \; & +\int_{0}^{t}\int_{|x|\geq 1}\left[f\left(X_{s-}+K\left(s,x\right)\right)-f\left(X_{s-}\right)\right]N\left(ds\;dx\right)\\ \; & +\int_{0}^{t}\int_{|x|<1}\left[f\left(X_{s-}+H\left(s,x\right)\right)-f\left(X_{s-}\right)\right]q\left(ds\;dx\right)\\ \; & +\int_{0}^{t}\int_{|x|<1}\left[f\left(X_{s-}+H\left(s,x\right)\right)-f\left(X_{s-}\right)-H\left(s,x\right)f^{\prime }\left(X_{s-}\right)\right]\bar{\nu }\left(dx\right)\;ds, \end{array} \end{array}$$

*where $X^{c}$ is the continuous part of X.*

### 2.4. Generalised Version of the Dupire Formula for the Margrabe Option

In this subsection, we present the generalisation of the Dupire formula, established in [14] for the Margrabe option in the Lévy-type stochastic integral setting.

All the stochastic processes and random variables used in this subsection are defined on a filtered probability space $\left(\Omega ,F,{\left(F_{t}\right)}_{t\in T},P\right)$ with $T=\left[0,T\right]$, T<∞, fulfilling the usual conditions. We additionally consider the associated risk-neutral probability measure Q equivalent to P.

A stochastic process $R_{t}=\exp\left(-\int_{0}^{t}r_{u}du\right),\;t\in T$, being a numeraire, describes the time value of money, where $r={\left(r_{t}\right)}_{t\in T}$ is the instantaneous risk-free interest rate.

We also consider stochastic processes $N={\left(N_{t}\right)}_{t\in T}$, $Y={\left(Y_{t}\right)}_{t\in T}$, $\tilde{N}={\left({\tilde{N}}_{t}\right)}_{t\in T}$, $\tilde{Y}={\left({\tilde{Y}}_{t}\right)}_{t\in T}$, and $S={\left(S_{t}\right)}_{t\in T}$, modelling the asset price, where $N_{t}>0$ a.s., t∈T, ${\tilde{N}}_{t}=N_{t}R_{t},\;{\tilde{Y}}_{t}=Y_{t}R_{t},\;S_{t}=Y_{t}/N_{t}={\tilde{Y}}_{t}/{\tilde{N}}_{t},\;t\in T.$ Thus, processes $\tilde{N}$ and $\tilde{Y}$ correspond to the discounted values of *N* and *Y*, respectively, and *S* models the “exchange rate” between them.

Moreover, in the approach presented in [14], $\tilde{N}={\left({\tilde{N}}_{t}\right)}_{t\in T}$ and $S={\left(S_{t}\right)}_{t\in T}$ are Lévy-type stochastic integrals of the form described with respect to Q by the following:

(6) $$\begin{array}{cc} \; & d{\tilde{N}}_{t}=\alpha_{t}dW_{t}^{\left(1\right)}+\beta_{t}dt+\int_{|y|<1}h_{1}\left(t,y\right)q\left(dt\;dy\right)+\int_{|y|\geq 1}k_{1}\left(t,y\right)N\left(dt\;dy\right),\;{\tilde{N}}_{0}>0, \end{array}$$

(7) $$\begin{array}{cc} \; & dS_{t}=\sigma_{t}dW_{t}^{\left(2\right)}+\gamma_{t}dt+\int_{|y|<1}h_{2}\left(t,y\right)q\left(dt\;dy\right)+\int_{|y|\geq 1}k_{2}\left(t,y\right)N\left(dt\;dy\right),\;S_{0}\in R_{+}, \end{array}$$

for the standard Brownian motions $W^{\left(i\right)}={\left(W_{t}^{\left(i\right)}\right)}_{t\in T},\;i\in \left\{1,2\right\}$ independent of N, with ${\left[W_{t}^{\left(1\right)},W_{t}^{\left(2\right)}\right]}_{t}=\rho t,\;t\in T,\left|\rho \right|\leq 1$, β,γ,α,σ∈M(T), $h_{1}1_{|y|<1}\left(y\right),h_{2}1_{|y|<1}\left(y\right),k_{1}1_{|y|\geq 1}\left(y\right)$, $k_{2}1_{|y|\geq 1}\left(y\right)\in M_{loc}^{2}\left(\hat{P},\bar{\nu },R\right)$, and X, N, *q*, ν, $\bar{\nu }$ described in Section 2.3 with respect to Q, and the following inequality holds.

(8) $$\begin{array}{cc} E^{Q}\int_{0}^{T} & \left[\alpha_{t}^{2}+\beta_{t}^{2}+\sigma_{t}^{2}+\gamma_{t}^{2}+\int_{|y|<1}\left(h_{1}^{4}\left(t,y\right)+h_{2}^{4}\left(t,y\right)\right)\bar{\nu }\left(dy\right)\right.\\ \; & \left.+\int_{|y|\geq 1}\left(k_{1}^{4}\left(t,y\right)+k_{2}^{4}\left(t,y\right)\right)\bar{\nu }\left(dy\right)+S_{t-}^{2}\left(1+\alpha_{t}^{2}+\int_{|y|<1}h_{1}^{2}\left(t,y\right)\bar{\nu }\left(dy\right)\right.\right.\\ \; & \left.\left.+\int_{|y|\geq 1}k_{1}^{2}\left(t,y\right)\bar{\nu }\left(dy\right)\right)+{\tilde{N}}_{t-}^{2}\left(1+S_{t-}^{2}+\sigma_{t}^{2}+\int_{|y|<1}h_{2}^{2}\left(t,y\right)\bar{\nu }\left(dy\right)\right.\right.\\ \; & \left.\left.+\int_{|y|\geq 1}k_{2}^{2}\left(t,y\right)\bar{\nu }\left(dy\right)\right)\right]dt<\infty . \end{array}$$

In the formula above, $E^{Q}$ denotes the expected value with respect to Q.

The differential of

(9) $${\tilde{Y}}_{t}={\tilde{N}}_{t}S_{t},\;t\in T,$$

with respect to Q is described by

$$d{\tilde{Y}}_{t}=\phi_{t}^{\left(1\right)}dW_{t}^{\left(1\right)}+\phi_{t}^{\left(2\right)}dW_{t}^{\left(2\right)}+\lambda_{t}dt+\int_{|y|<1}\xi^{\left(1\right)}\left(t,y\right)q\left(dt\;dy\right)+\int_{|y|\geq 1}\xi^{\left(2\right)}\left(t,y\right)N\left(dt\;dy\right),$$

where

$$\begin{array}{cc} \; & \phi_{t}^{\left(1\right)}=S_{t-}\alpha_{t},\;\phi_{t}^{\left(2\right)}={\tilde{N}}_{t-}\sigma_{t},\;\lambda_{t}={\tilde{N}}_{t-}\gamma_{t}+S_{t-}\beta_{t}+\rho \alpha_{t}\sigma_{t}+\int_{|y|<1}h_{1}\left(t,y\right)h_{2}\left(t,y\right)\bar{\nu }\left(dy\right),\\ \; & \xi^{\left(1\right)}\left(t,y\right)={\tilde{N}}_{t-}h_{2}\left(t,y\right)+S_{t-}h_{1}\left(t,y\right)+h_{1}\left(t,y\right)h_{2}\left(t,y\right),\\ \; & \xi^{\left(2\right)}\left(t,y\right)={\tilde{N}}_{t-}k_{2}\left(t,y\right)+S_{t-}k_{1}\left(t,y\right)+k_{1}\left(t,y\right)k_{2}\left(t,y\right). \end{array}$$

We will also use the following auxiliary functions.

$$\begin{array}{cc} \; & {\tilde{h}}_{1}\left(t,y\right)=h_{1}\left(t,y\right)1_{|y|<1}\left(y\right),{\tilde{h}}_{2}\left(t,y\right)=h_{2}\left(t,y\right)1_{|y|<1}\left(y\right),\;{\tilde{k}}_{1}\left(t,y\right)=k_{1}\left(t,y\right)1_{|y|\geq 1}\left(y\right),\\ \; & {\tilde{k}}_{2}\left(t,y\right)=k_{2}\left(t,y\right)1_{|y|\geq 1}\left(y\right),\;{\tilde{\xi }}^{\left(2\right)}\left(t,y\right)=\xi^{\left(2\right)}\left(t,y\right)1_{|y|\geq 1}\left(y\right). \end{array}$$

It was proved in [14] that the integrals describing $\tilde{Y}$ are well defined.

According to the theory of pricing financial derivatives, it follows that the no-arbitrage time-zero price of a contingent claim *H* is equal to the expected value with respect to Q of its pay-off discounted by $R_{t}$. *H*, in the considered case of the Margrabe option, is regarded as the European option to exchange the asset with the price modelled by *Y* for *k* units of the asset described by the price process *N*. We assume the existence of the risk-neutral probability measure Q, and therefore, the no-arbitrage price $c\left(t,k\right)$ at time zero of the contingent claim *H* with maturity t∈T is given by the following:

(10) $$\begin{array}{cc} \; & c\left(t,k\right)=E^{Q}\left[R_{t}{\left(Y_{t}-kN_{t}\right)}_{+}\right]=E^{Q}\left[{\left({\tilde{Y}}_{t}-k{\tilde{N}}_{t}\right)}_{+}\right]\\ \; & \;\;=E^{Q}\left[R_{t}N_{t}{\left(S_{t}-k\right)}_{+}\right]=E^{Q}\left[{\tilde{N}}_{t}{\left(S_{t}-k\right)}_{+}\right],\;k\in R. \end{array}$$

By [14] (Lemma 3.1), the function $c:R^{2}\to R$ of the form

$$c\left(t,k\right)=c\left(t,k\right)1_{T\times R}\left(t,k\right)$$

belongs to $L_{loc}^{1}\left(R^{2}\right)$, and therefore, $C=\iota_{\left(0,T\right)\times R,R^{2}}\left(c\right)$ is an element of $D^{\prime }$.

Let $l\left(dt\right)=dt$ denote the Lebesgue measure on the σ-algebra $L\left(T\right)$ of Lebesgue subsets of T.

The generalised Dupire formula, derived and proved in [14], is formulated in the following theorem.

**Theorem** **2.** *Under the assumptions that the stochastic processes $\tilde{N}={\left({\tilde{N}}_{t}\right)}_{t\in T}$, $S={\left(S_{t}\right)}_{t\in T}$, and $\tilde{Y}={\left({\tilde{Y}}_{t}\right)}_{t\in T}$ are given by (6), (7), and (9) and the inequality (8) is satisfied,*

(11) $$\begin{array}{c} \begin{array}{cc} \frac{\partial C}{\partial t}= & \frac{1}{2}E^{t}\left(\sigma_{t}^{2}|S_{t-}=k\right)\frac{\partial^{2}C}{\partial k^{2}}+\int_{k}^{\infty }E^{t}\left(\left.\frac{\lambda_{t}}{{\tilde{N}}_{t-}}-\frac{\beta_{t}}{{\tilde{N}}_{t-}}k\right|S_{t-}=x\right)\Phi \left(t,dx\right)\\ \; & +\int_{R}\int_{k}^{\infty }E^{t}\left(\left.\frac{{\tilde{\xi }}^{\left(2\right)}\left(t,y\right)}{{\tilde{N}}_{t-}}-\frac{{\tilde{k}}_{1}\left(t,y\right)}{{\tilde{N}}_{t-}}k\right|S_{t-}=x\right)\Phi \left(t,dx\right)\bar{\nu }\left(dy\right)\\ \; & +\int_{R}\int_{R}E^{t}\left(\frac{{\tilde{h}}_{1}\left(t,y\right)j\left(S_{t-},{\tilde{h}}_{2}\left(t,y\right)\right)\left(k\right)+{\tilde{k}}_{1}\left(t,y\right)j\left(S_{t-},{\tilde{k}}_{2}\left(t,y\right)\right)\left(k\right)}{{\tilde{N}}_{t-}}\right.\\ \; & \;\;\left.\left.+j\left(S_{t-},{\tilde{h}}_{2}\left(t,y\right)\right)\left(k\right)+j\left(S_{t-},{\tilde{k}}_{2}\left(t,y\right)\right)\left(k\right)\right|S_{t-}=x\right)\Phi \left(t,dx\right)\bar{\nu }\left(dy\right) \end{array} \end{array}$$

*in $D^{\prime }$. In the equation above, Φ(t,dx) is the finite transition kernel from $\left(T,L\left(T\right)\right)$ to $\left(R,B\left(R\right)\right)$ given by (12), and $\frac{\partial^{2}C}{\partial k^{2}}=l⊗\Phi$ is interpreted as the finite measure on $\left(T\times R,L\left(T\right)⊗B\left(R\right)\right)$ such that (13) holds. We also use the notation $E^{t}$ for the expected values with respect to the probability measures $Q^{t}$ defined by the formula*

$$Q^{t}\left(A\right)=E^{Q}\left({\tilde{N}}_{t-}1_{A}\right)/E^{Q}{\tilde{N}}_{t-},\;A\in F,\;t\in T.$$

For the probability distribution $\mu_{S_{t-}}^{t}$ of $S_{t-}$ with respect to $Q^{t}$, the following equality in $D^{\prime }$ is fulfilled:

$$\begin{array}{cc} \; & \frac{\partial C}{\partial k}=-E^{Q}{\tilde{N}}_{t-}E^{t}1_{S_{t-}>k}, \end{array}$$

and

(12) $$\Phi \left(t,dx\right)=E^{Q}{\tilde{N}}_{t-}\mu_{S_{t-}}^{t}\left(dx\right)$$

represents a transition kernel from $\left(T,L\left(T\right)\right)$ to $\left(R,B\left(R\right)\right)$. Moreover,

(13) $$l⊗\Phi \left(A_{1}\times A_{2}\right)=\int_{A_{1}}\Phi \left(t,A_{2}\right)dt,\;A_{1}\in L\left(T\right),\;A_{2}\in B\left(R\right),$$

describes a finite measure on $\left(T\times R,L\left(T\right)⊗B\left(R\right)\right)$ and we have $\frac{\partial^{2}C}{\partial k^{2}}=l⊗\Phi$.

**Remark** **1.** *The Dupire Equation (*11*) in $D^{\prime }$ can be formulated as follows:*

$$\begin{array}{c} \begin{array}{cc} \; & -\int_{0}^{T}\int_{-\infty }^{\infty }\;f_{t}^{\prime }\left(t,k\right)C\left(t,k\right)dk\;dt\\ \; & =\frac{1}{2}\int_{0}^{T}\int_{-\infty }^{\infty }f\left(t,k\right)E^{t}\left(\sigma_{t}^{2}|S_{t-}=k\right)\frac{\partial^{2}C}{\partial k^{2}}\left(dt\;dk\right)\\ \; & +\int_{0}^{T}\int_{-\infty }^{\infty }f\left(t,k\right)\int_{k}^{\infty }E^{t}\left(\left.\frac{\lambda_{t}}{{\tilde{N}}_{t-}}-\frac{\beta_{t}}{{\tilde{N}}_{t-}}k\right|S_{t-}=x\right)\Phi \left(t,dx\right)\;dk\;dt\\ \; & +\int_{0}^{T}\int_{-\infty }^{\infty }f\left(t,k\right)\int_{R}\int_{k}^{\infty }E^{t}\\ \; & \;\;\;\;\;\;\;\;\;\;\;\;\left(\left.\frac{{\tilde{\xi }}^{\left(2\right)}\left(t,y\right)}{{\tilde{N}}_{t-}}-\frac{{\tilde{k}}_{1}\left(t,y\right)}{{\tilde{N}}_{t-}}k\right|S_{t-}=x\right)\Phi \left(t,dx\right)\bar{\nu }\left(dy\right)\;dk\;dt\\ \; & +\int_{0}^{T}\int_{-\infty }^{\infty }f\left(t,k\right)\int_{R}\int_{R}E^{t}\\ \; & \;\;\;\;\;\;\;\;\;\;\;\;\left(\frac{{\tilde{h}}_{1}\left(t,y\right)j\left(S_{t-},{\tilde{h}}_{2}\left(t,y\right)\right)\left(k\right)+{\tilde{k}}_{1}\left(t,y\right)j\left(S_{t-},{\tilde{k}}_{2}\left(t,y\right)\right)\left(k\right)}{{\tilde{N}}_{t-}}\right.\\ \; & \;\;\;\;\;\;\;\;\;\;\;\;\;\left.\left.+j\left(S_{t-},{\tilde{h}}_{2}\left(t,y\right)\right)\left(k\right)+j\left(S_{t-},{\tilde{k}}_{2}\left(t,y\right)\right)\left(k\right)\right|S_{t-}=x\right)\Phi \left(t,dx\right)\bar{\nu }\left(dy\right)\;dk\;dt \end{array} \end{array}$$

*for an arbitrary R-valued $C^{\infty }$- function f on $R^{2}$ of compact support in $\left(0,T\right)\times R$.*

## 3. The Dupire Formula for the Merton Model with Stochastic Interest Rate

This section is devoted to deriving the Dupire formula for the Merton model (1976) (see [35]) of the underlying asset with stochastic interest rate. To this end, we apply Theorem 2 in the case of the mentioned model. We also use the theory of pricing contingent claims and the theory from [21], and we adapt the form of the equation of the underlying asset formulated in [7] (Eqn. (1)) to our setting (see (21)).

We denote by the symbol $r={\left(r_{t}\right)}_{t\in T}$ the risk-free instantaneous interest rate (or spot interest rate), described by an adapted stochastic process with sample paths integrable on T with respect to the Lebesgue measure l(dt)=dt a.s., by the symbol $B={\left(B_{t}\right)}_{t\in T}$ an adapted process of finite variation and with continuous sample paths of the form

$$B_{t}=\exp\left(\int_{0}^{t}r_{u}du\right),\;t\in T,$$

called *a savings account*, and by the symbol B(s,t), 0≤s≤t≤T, adapted processes, where B(s,t) the price at time *s* of a zero-coupon bond of maturity *t*.

We assume that B(s,t), 0≤s≤t≤T, is an arbitrage-free family of bond prices relative to *r*, i.e., the following conditions hold (see [21] (Def. 9.5.1)):

(a) B(t,t)=1, t∈T.
(b) There exists a probability measure $P^{*}$ on $\left(\Omega ,F_{T}\right)$ (called *a spot martingale measure for the family* B(s,t)) equivalent to P such that for each t∈T process $B\left(s,t\right)/B_{s}$, s∈[0,t], is a $P^{*}$-martingale.

The existence of a spot martingale measure $P^{*}$ for the family B(s,t) implies the absence of arbitrage opportunities between bonds and a savings account (see [21]). Moreover, the following equality holds (see [21] (Eqn. 9.14))

(14) $$B\left(s,t\right)=E^{*}\left(\exp\left(-\int_{s}^{t}r_{u}du\right)|F_{s}\right),\;0\leq s\leq t\leq T,$$

where $E^{*}$ is the expectation with respect to $P^{*}$. To preclude the possibility of arbitrage, we will also assume that the considered asset price discounted by *B* is a Q-martingale for $Q=P^{*}$.

Apart from a spot martingale measure, we will also consider, for t∈T, a probability measure $P_{t}$ on $\left(\Omega ,F_{t}\right)$ equivalent to $P^{*}$ (called the forward martingale measure for the settlement date *t*), described by the Radon–Nikodým derivative as follows (see [21] (Def. 9.6.2))

(15) $$\frac{dP_{t}}{dP^{*}}=\frac{1}{B\left(0,t\right)B_{t}},\;P^{*}-a.s.$$

The expected value with respect to $P_{t}$ will be denoted by $E^{P_{t}}$.

We will assume that with respect to the probability measure $Q=P^{*}$, the price process *S* and the discounted price process $\tilde{S}$ of the underlying asset take the form

$$\begin{array}{cc} S_{t}= & S_{0}\exp\left(\int_{0}^{t}{\overset{ˇ}{\mu }}_{u}du+\int_{0}^{t}\sigma_{u}dW_{u}^{\left(2\right)}+\int_{0}^{t}\int_{R}yN\left(du\;dy\right)\right),\;t\in T,\\ {\tilde{S}}_{t}= & \exp\left(-\int_{0}^{t}r_{u}du\right)S_{0}\exp\left(\int_{0}^{t}{\overset{ˇ}{\mu }}_{u}du+\int_{0}^{t}\sigma_{u}dW_{u}^{\left(2\right)}+\int_{0}^{t}\int_{R}yN\left(du\;dy\right)\right)\\ = & S_{0}\exp\left(\int_{0}^{t}\left({\overset{ˇ}{\mu }}_{u}-r_{u}\right)du+\int_{0}^{t}\sigma_{u}dW_{u}^{\left(2\right)}+\int_{0}^{t}\int_{R}yN\left(du\;dy\right)\right),\;t\in T, \end{array}$$

respectively, for sufficiently regular parameters. Then, by Theorem 1

(16) $$\begin{array}{cc} dS_{t} & =S_{t-}\left(\left({\overset{ˇ}{\mu }}_{t}+\frac{\sigma_{t}^{2}}{2}+\int_{R}\left(e^{y}-1\right)\bar{\nu }\left(dy\right)\right)dt+\sigma_{t}dW_{t}^{\left(2\right)}\right. \end{array}$$

(17) $$\begin{array}{cc} \; & \left.+\int_{R}\left(e^{y}-1\right)q\left(dt\;dy\right)\right),\\ d{\tilde{S}}_{t} & ={\tilde{S}}_{t-}\left(\left(-r_{t}+{\overset{ˇ}{\mu }}_{t}+\frac{\sigma_{t}^{2}}{2}+\int_{R}\left(e^{y}-1\right)\bar{\nu }\left(dy\right)\right)dt+\sigma_{t}dW_{t}^{\left(2\right)}\right.\\ \; & \left.+\int_{R}\left(e^{y}-1\right)q\left(dt\;dy\right)\right). \end{array}$$

To ensure that $\tilde{S}$ is a Q- martingale, we should have

$${\overset{ˇ}{\mu }}_{t}=r_{t}-\frac{\sigma_{t}^{2}}{2}-\int_{R}\left(e^{y}-1\right)\bar{\nu }\left(dy\right).$$

Thus,

$$\begin{array}{cc} dS_{t}= & S_{t-}\left(r_{t}dt+\sigma_{t}dW_{t}^{\left(2\right)}+\int_{R}\left(e^{y}-1\right)q\left(dt\;dy\right)\right) \end{array}$$

and for sufficiently regular parameters, $\tilde{S}$ is the solution of the equation

(18) $$\begin{array}{cc} d{\tilde{S}}_{t}= & {\tilde{S}}_{t-}\left(\sigma_{t}dW_{t}^{\left(2\right)}+\int_{R}\left(e^{y}-1\right)q\left(dt\;dy\right)\right). \end{array}$$

Therefore, *S* can be written in the form

(19) $$\begin{array}{cc} S_{t}= & B_{t}{\tilde{S}}_{t},\;t\in T, \end{array}$$

where $\tilde{S}$ is the solution of (18).

**Theorem** **3.** *Let, under Q, the instantaneous interest rate $r={\left\{r_{t}\right\}}_{t\in T}$ and the underlying asset price $S={\left\{S_{t}\right\}}_{t\in T}$ be described by the Merton model of 1973 and 1976, respectively,*

(20) $$\begin{array}{cc} \; & r_{t}=r_{0}+\mu_{r}t+\sigma_{r}W_{t}^{\left(1\right)},\;t\in T, \end{array}$$

(21) $$\begin{array}{cc} \; & N_{t}\equiv 1,\;\;{\tilde{N}}_{t}=R_{t}=exp\left(-\int_{0}^{t}r_{u}du\right),\;t\in T,\\ \; & S_{t}=S_{0}+\int_{0}^{t}{\overset{ˇ}{r}}_{u}S_{u-}du+\int_{0}^{t}\bar{\sigma }\left(u,S_{u-}\right)dW_{u}^{\left(2\right)}+\int_{0}^{t}\int_{|y|<1}\left(e^{y}-1\right)S_{u-}q\left(du\;dy\right)\\ \; & \;\;\;\;\;\;+\int_{0}^{t}\int_{|y|\geq 1}\left(e^{y}-1\right)S_{u-}N\left(du\;dy\right),\;t\in T,\\ \; & {\overset{ˇ}{r}}_{t}=r_{t}-\overset{ˇ}{\gamma },\;\overset{ˇ}{\gamma }=\int_{|y|\geq 1}\left(e^{y}-1\right)\bar{\nu }\left(dy\right),\\ \; & \bar{\sigma }\left(t,k\right)=\overset{=}{\sigma }\left(t,k\right)k,\;\;\left(t,k\right)\in T\times R, \end{array}$$

*where the real numbers $S_{0},\;r_{0},\mu_{r},$ and $\;\sigma_{r}$ satisfy the inequalities $S_{0},r_{0},\mu_{r},\sigma_{r}>0$, $\bar{\sigma },\overset{=}{\sigma }:T\times R\to R$ are continuous functions ($\overset{=}{\sigma }$ is called the local volatility),*

*(i)*  *For l-a.e. t∈T, $k\in R\;\;\;\;\;\;\;\;\;\;\;\left|\bar{\sigma }\left(t,k\right)\right|\leq C_{G}\left(1+\left|k\right|\right)$.*
*(ii)*  *For l-a.e. t∈T, $k_{1},k_{2}\in R$    $\left|\bar{\sigma }\left(t,k_{1}\right)-\bar{\sigma }\left(t,k_{2}\right)\right|\leq C_{L}\left|k_{1}-k_{2}\right|$.*

*$C_{G},\;C_{L}>0$, N is the Poisson random measure associated with the Lévy process, which is the compound Poisson process ${\left(X_{t}\right)}_{t\in T}$ independent of W; X has a constant rate $\overset{ˇ}{\lambda }>0$; the normal distribution $\overset{ˇ}{\varphi }$ of jumps with the mean $\overset{ˇ}{\mu }$ and variance ${\overset{ˇ}{\gamma }}^{2}$; and $\bar{\nu }\left(dx\right)=\overset{ˇ}{\lambda }\overset{ˇ}{\varphi }\left(dx\right)$. Then, the Dupire formula for C has the following form in $D^{\prime }$:*

(22) $$\begin{array}{c} \begin{array}{cc} \frac{\partial C}{\partial t}= & \frac{{\overset{=}{\sigma }}^{2}\left(t,k\right)k^{2}}{2}\frac{\partial^{2}C}{\partial k^{2}}+kB\left(0,t\right)E^{P_{t}}\left(r_{t}1_{S_{t-}>k}\right)\\ \; & +\int_{R}e^{y}\left(C\left(t,ke^{-y}\right)-C\left(t,k\right)-\left(e^{-y}-1\right)k\frac{\partial C\left(t,k\right)}{\partial k}\right)\overset{ˇ}{\lambda }\overset{ˇ}{\varphi }\left(dy\right). \end{array} \end{array}$$

**Proof.**  By [21] (p. 385) for t∈T, $B_{t}=e^{{\overset{ˇ}{\xi }}_{t}}$, where

(23) $$\begin{array}{c} {\overset{ˇ}{\xi }}_{t}=r_{0}t+\frac{1}{2}\mu_{r}t^{2}+\int_{0}^{t}\sigma_{r}\left(t-u\right)dW_{u}^{\left(1\right)}, \end{array}$$

${\overset{ˇ}{\xi }}_{t}$ has a normal distribution with the expected value $r_{0}t+\frac{1}{2}\mu_{r}t^{2}$ and the variance $\frac{1}{3}\sigma_{r}^{2}t^{3}$. As stated in [36], if a random variable *V* has a normal distribution with the expected value $\mu_{v}$ and the variance $\sigma_{v}^{2}$, then, $\bar{V}=e^{V}$ has a log-normal distribution with moments given by the formula

(24) $$E^{Q}{\bar{V}}^{q}=\exp\left(q\mu_{v}+\frac{1}{2}q^{2}\sigma_{v}^{2}\right),\;q\in N$$

and therefore, for q∈N and t∈T,

(25) $$E^{Q}B_{t}^{q}=\exp\left(q\left(r_{0}t+\frac{1}{2}\mu_{r}t^{2}\right)+\frac{1}{6}q^{2}\sigma_{r}^{2}t^{3}\right)$$

and

(26) $$E^{Q}{\tilde{N}}_{t}^{q}=\exp\left(q\left(-r_{0}t-\frac{1}{2}\mu_{r}t^{2}\right)+\frac{1}{6}q^{2}\sigma_{r}^{2}t^{3}\right).$$

Thus, for q∈N and t∈T,

(27) $$E^{Q}B_{t}^{q}\leq b_{q}:=\exp\left(q\left(r_{0}T+\frac{1}{2}\mu_{r}T^{2}\right)+\frac{1}{6}q^{2}\sigma_{r}^{2}T^{3}\right)<\infty$$

and

(28) $$E^{Q}{\tilde{N}}_{t}^{q}\leq n_{q}:=\exp\left(\frac{1}{6}q^{2}\sigma_{r}^{2}T^{3}\right)<\infty .$$

By [37] (Thm. 3.1) and conditions (i) and (ii), Equation (18) for $\sigma_{t}=\overset{=}{\sigma }\left(t,S_{t-}\right),\;t\in T$, has a unique solution and

(29) $$m_{q}=E^{Q}{\left({\tilde{S}}_{T}^{*}\right)}^{q}<\infty ,\;q\geq 2,$$

where ${\tilde{S}}_{T}^{*}:=\sup_{t\in T}{\tilde{S}}_{t}$. Therefore, by Hölder’s inequality

(30) $$\begin{array}{cc} \int_{0}^{T}E^{Q}S_{t}^{q}dt & \leq \int_{0}^{T}E^{Q}{\left(\underset{t\in T}{\sup}{\tilde{S}}_{t}B_{t}\right)}^{q}dt\leq \int_{0}^{T}{\left(E^{Q}{\left(\underset{t\in T}{\sup}{\tilde{S}}_{t}\right)}^{2q}\right)}^{\frac{1}{2}}{\left(E^{Q}B_{t}^{2q}\right)}^{\frac{1}{2}}dt\\ \; & \leq m_{2q}^{\frac{1}{2}}b_{2q}^{\frac{1}{2}}T<\infty . \end{array}$$

(31) $$\begin{array}{cc} \int_{0}^{T}{\left(E^{Q}S_{t}^{q}\right)}^{\frac{1}{2}}dt & \leq \int_{0}^{T}{\left(E^{Q}{\left(\underset{t\in T}{\sup}{\tilde{S}}_{t}B_{t}\right)}^{q}\right)}^{\frac{1}{2}}dt\leq \int_{0}^{T}{\left(E^{Q}{\left(\underset{t\in T}{\sup}{\tilde{S}}_{t}\right)}^{2q}\right)}^{\frac{1}{4}}{\left(E^{Q}B_{t}^{2q}\right)}^{\frac{1}{4}}dt\\ \; & \leq m_{2q}^{\frac{1}{4}}b_{2q}^{\frac{1}{4}}T<\infty . \end{array}$$

We also have

(32) $$\begin{array}{cc} E^{Q}r_{t}^{4} & \leq E^{Q}{\left(2{\left(r_{0}+\mu_{r}t\right)}^{2}+2{\left(\sigma_{r}W_{t}^{\left(1\right)}\right)}^{2}\right)}^{2}\leq E^{Q}\left(8{\left(r_{0}+\mu_{r}t\right)}^{4}+8{\left(\sigma_{r}W_{t}^{\left(1\right)}\right)}^{4}\right)\\ \; & =8{\left(r_{0}+\mu_{r}t\right)}^{4}+24\sigma_{r}^{4}t^{2}\leq c_{r}:=8{\left(r_{0}+\mu_{r}T\right)}^{4}+24\sigma_{r}^{4}T^{2}<\infty \end{array}$$

and

(33) $$\begin{array}{cc} E^{Q}{\overset{ˇ}{r}}_{t}^{4} & \leq E^{Q}{\left(2{\left(r_{0}-\overset{ˇ}{\gamma }+\mu_{r}t\right)}^{2}+2{\left(\sigma_{r}W_{t}^{\left(1\right)}\right)}^{2}\right)}^{2}\leq E^{Q}\left(8{\left(r_{0}-\overset{ˇ}{\gamma }+\mu_{r}t\right)}^{4}\right.\\ & \left.+8{\left(\sigma_{r}W_{t}^{\left(1\right)}\right)}^{4}\right)=8{\left(r_{0}+\mu_{r}t-\overset{ˇ}{\gamma }\right)}^{4}+24\sigma_{r}^{4}t^{2}\leq 32{\left({\left(r_{0}+\mu_{r}t\right)}^{2}+{\overset{ˇ}{\gamma }}^{2}\right)}^{2}+24\sigma_{r}^{4}t^{2}\\ \; & \leq c_{\overset{ˇ}{r}}:=32{\left({\left(r_{0}+\mu_{r}T\right)}^{2}+{\overset{ˇ}{\gamma }}^{2}\right)}^{2}+24\sigma_{r}^{4}T^{2}<\infty . \end{array}$$

Moreover, there exists $C_{E}>0$, such that

(34) $$\int_{R}{\left(e^{y}-1\right)}^{2}\bar{\nu }\left(dx\right),\int_{R}{\left(e^{y}-1\right)}^{4}\bar{\nu }\left(dx\right)\leq C_{E},$$

since these integrals are finite. We also have $\beta_{t}=-r_{t}{\tilde{N}}_{t}$ by Theorem 1.

And

$$\begin{array}{cc} \; & \alpha ={\tilde{h}}_{1}={\tilde{k}}_{1}=\phi^{\left(1\right)}=0,\;\sigma_{t}=\bar{\sigma }\left(t,S_{t-}\right),\;\gamma_{t}={\overset{ˇ}{r}}_{t}S_{t-},\\ \; & {\tilde{h}}_{2}\left(t,y\right)=\left(e^{y}-1\right)1_{|y|<1}S_{t-},{\tilde{k}}_{2}\left(t,y\right)=\left(e^{y}-1\right)1_{|y|\geq 1}S_{t-},\;\phi_{t}^{\left(2\right)}={\tilde{N}}_{t-}\sigma_{t}={\tilde{N}}_{t-}\bar{\sigma }\left(t,S_{t-}\right),\\ \; & \lambda_{t}=-\overset{ˇ}{\gamma }{\tilde{N}}_{t-}S_{t-},\;{\tilde{\xi }}^{\left(2\right)}\left(t\right)=\left(e^{y}-1\right)1_{|y|\geq 1}{\tilde{N}}_{t-}S_{t-},\;Q^{t}=P^{t},\;t\in T. \end{array}$$

We also have for t∈T,

$$\begin{array}{cc} \; & E^{t}\left(\sigma_{t}^{2}|S_{t-}=k\right)=E^{Q}\left(\sigma_{t}^{2}|S_{t-}=k\right)=E^{Q}\left({\bar{\sigma }}^{2}\left(t,S_{t}\right)|S_{t-}=k\right)={\bar{\sigma }}^{2}\left(t,k\right),\\ \; & E^{t}\left(\left.\frac{\lambda_{t}}{{\tilde{N}}_{t-}}-\frac{\beta_{t}}{{\tilde{N}}_{t-}}k\right|S_{t-}=x\right)=E^{Q}\left(\left.-\overset{ˇ}{\gamma }S_{t-}+\frac{r_{t}{\tilde{N}}_{t-}}{{\tilde{N}}_{t-}}k\right|S_{t-}=x\right)=-\overset{ˇ}{\gamma }x\\ \; & +kE^{Q}\left(\left.r_{t}\right|S_{t-}=x\right),\\ \; & E^{t}\left(\left.\frac{{\tilde{\xi }}^{\left(2\right)}\left(t,y\right)}{{\tilde{N}}_{t-}}-\frac{{\tilde{k}}_{1}\left(t,y\right)}{{\tilde{N}}_{t-}}k\right|S_{t-}=x\right)=E^{t}\left(\left.\frac{\left(e^{y}-1\right)1_{|y|\geq 1}{\tilde{N}}_{t-}S_{t-}}{{\tilde{N}}_{t-}}\right|S_{t-}=x\right)\\ \; & =\left(e^{y}-1\right)1_{|y|\geq 1}x,\\ \; & E^{t}\left(\frac{{\tilde{h}}_{1}\left(t,y\right)j\left(S_{t-},{\tilde{h}}_{2}\left(t,y\right)\right)\left(k\right)+{\tilde{k}}_{1}\left(t,y\right)j\left(S_{t-},{\tilde{k}}_{2}\left(t,y\right)\right)\left(k\right)}{{\tilde{N}}_{t-}}\right.\\ \; & \;\;\;\;\;\;\;\;\;\left.\left.+j\left(S_{t-},{\tilde{h}}_{2}\left(t,y\right)\right)\left(k\right)+j\left(S_{t-},{\tilde{k}}_{2}\left(t,y\right)\right)\left(k\right)\right|S_{t-}=x\right)\\ \; & \;\;\;\;\;\;\;\;\;=E^{t}\left(\left.j\left(S_{t-},{\tilde{h}}_{2}\left(t,y\right)\right)\left(k\right)+j\left(S_{t-},{\tilde{k}}_{2}\left(t,y\right)\right)\left(k\right)\right|S_{t-}=x\right)\\ \; & \;\;\;\;\;\;\;\;\;=E^{t}\left(\left.{\left(S_{t-}e^{y}-k\right)}_{+}-{\left(S_{t-}-k\right)}_{+}-S_{t-}\left(e^{y}-1\right)g_{S_{t-}}\left(k\right)\right|S_{t-}=x\right)\\ \; & \;\;\;\;\;\;\;\;\;=E^{t}\left(\left.{\left(S_{t-}e^{y}-k\right)}_{+}-{\left(S_{t-}-k\right)}_{+}-\left(e^{y}-1\right)\left[{\left(S_{t-}-k\right)}_{+}+k\right]g_{S_{t-}}\left(k\right)\right|S_{t-}=x\right)\\ \; & \;\;\;\;\;\;\;\;\;=e^{y}\left({\left(x-ke^{-y}\right)}_{+}-{\left(x-k\right)}_{+}-\left(1-e^{-y}\right)kg_{x}\left(k\right)\right),\\ \; & \int_{k}^{\infty }\Phi \left(t,dx\right)=E^{Q}{\tilde{N}}_{t-}E^{t}1_{S_{t-}>k}=-\frac{\partial C\left(t,k\right)}{\partial k}, \end{array}$$

$$\begin{array}{cc} \; & \int_{k}^{\infty }E^{t}\left(\left.\frac{\lambda_{t}}{{\tilde{N}}_{t-}}-\frac{\beta_{t}}{{\tilde{N}}_{t-}}k\right|S_{t-}=x\right)\Phi \left(t,dx\right)=\int_{k}^{\infty }\left(-\overset{ˇ}{\gamma }x\right)\Phi \left(t,dx\right)\\ \; & +k\int_{R}1_{x>k}E^{t}\left(\left.r_{t}\right|S_{t-}=x\right)\Phi \left(t,dx\right)=\int_{k}^{\infty }\left(-\overset{ˇ}{\gamma }x\right)\Phi \left(t,dx\right)\\ \; & +k\int_{R}E^{t}\left(\left.r_{t}1_{S_{t-}>k}\right|S_{t-}=x\right)\Phi \left(t,dx\right)=-\overset{ˇ}{\gamma }E^{Q}{\tilde{N}}_{t-}\int_{k}^{\infty }x\mu_{S_{t-}}^{t}\left(dx\right)\\ \; & +kE^{Q}{\tilde{N}}_{t-}E^{t}\left(r_{t}1_{S_{t-}>k}\right)=-\overset{ˇ}{\gamma }E^{Q}{\tilde{N}}_{t-}\int_{k}^{\infty }x\mu_{S_{t-}}^{t}\left(dx\right)+kB\left(0,t\right)E^{P_{t}}\left(r_{t}1_{S_{t-}>k}\right),\\ \; & \int_{R}\int_{k}^{\infty }E^{t}\left(\left.\frac{{\tilde{\xi }}^{\left(2\right)}\left(t,y\right)}{{\tilde{N}}_{t-}}-\frac{{\tilde{k}}_{1}\left(t,y\right)}{{\tilde{N}}_{t-}}k\right|S_{t-}=x\right)\Phi \left(t,dx\right)\bar{\nu }\left(dy\right)\\ \; & =\int_{R}\int_{k}^{\infty }\left(e^{y}-1\right)1_{|y|\geq 1}x\Phi \left(t,dx\right)\bar{\nu }\left(dy\right)=\overset{ˇ}{\gamma }E^{Q}{\tilde{N}}_{t-}\int_{k}^{\infty }x\mu_{S_{t-}}^{t}\left(dx\right), \end{array}$$

and, similarly to [9,14],

$$\begin{array}{cc} \; & \int_{R}\int_{R}E^{t}\left(\frac{{\tilde{h}}_{1}\left(t,y\right)j\left(S_{t-},{\tilde{h}}_{2}\left(t,y\right)\right)\left(k\right)+{\tilde{k}}_{1}\left(t,y\right)j\left(S_{t-},{\tilde{k}}_{2}\left(t,y\right)\right)\left(k\right)}{{\tilde{N}}_{t-}}\right.\\ \; & \;\;\;\;\;\;\;\;\;\left.\left.+j\left(S_{t-},{\tilde{h}}_{2}\left(t,y\right)\right)\left(k\right)+j\left(S_{t-},{\tilde{k}}_{2}\left(t,y\right)\right)\left(k\right)\right|S_{t-}=x\right)\Phi \left(t,dx\right)\bar{\nu }\left(dy\right)\\ \; & \;\;\;\;\;\;\;\;\;=\int_{R}\int_{R}e^{y}\left({\left(x-ke^{-y}\right)}_{+}-{\left(x-k\right)}_{+}-\left(1-e^{-y}\right)kg_{x}\left(k\right)\right)\Phi \left(t,dx\right)\bar{\nu }\left(dy\right)\\ \; & \;\;\;\;\;\;\;\;\;=\int_{R}e^{y}E^{Q}{\tilde{N}}_{t-}\int_{R}\left({\left(x-ke^{-y}\right)}_{+}-{\left(x-k\right)}_{+}-\left(1-e^{-y}\right)kg_{x}\left(k\right)\right)\mu_{S_{t-}}^{t}\left(dx\right)\bar{\nu }\left(dy\right)\\ \; & \;\;\;\;\;\;\;\;\;=\int_{R}e^{y}\left(C\left(t,ke^{-y}\right)-C\left(t,k\right)-E^{Q}{\tilde{N}}_{t-}\left(1-e^{-y}\right)k\int_{k}^{\infty }\mu_{S_{t-}}^{t}\left(dx\right)\right)\bar{\nu }\left(dy\right)\\ \; & \;\;\;\;\;\;\;\;\;=\int_{R}e^{y}\left(C\left(t,ke^{-y}\right)-C\left(t,k\right)-\left(e^{-y}-1\right)k\frac{\partial C\left(t,k\right)}{\partial k}\right)\overset{ˇ}{\lambda }\overset{ˇ}{\varphi }\left(dy\right). \end{array}$$

Additionally, by (i), (27)–(34),

$$\begin{array}{cc} E^{Q}\int_{0}^{T} & \left[\alpha_{t}^{2}+\beta_{t}^{2}+\sigma_{t}^{2}+\gamma_{t}^{2}+\int_{|x|<1}\left(h_{1}^{4}\left(t,x\right)+h_{2}^{4}\left(t,x\right)\right)\bar{\nu }\left(dx\right)\right.\\ \; & \left.+\int_{|x|\geq 1}\left(k_{1}^{4}\left(t,x\right)+k_{2}^{4}\left(t,x\right)\right)\bar{\nu }\left(dx\right)+S_{t-}^{2}\left(1+\alpha_{t}^{2}+\int_{|x|<1}h_{1}^{2}\left(t,x\right)\bar{\nu }\left(dx\right)\right.\right.\\ \; & \left.\left.+\int_{|x|\geq 1}k_{1}^{2}\left(t,x\right)\bar{\nu }\left(dx\right)\right)+{\tilde{N}}_{t-}^{2}\left(1+S_{t-}^{2}+\sigma_{t}^{2}+\int_{|x|<1}h_{2}^{2}\left(t,x\right)\bar{\nu }\left(dx\right)\right.\right.\\ \; & \left.\left.+\int_{|x|\geq 1}k_{2}^{2}\left(t,x\right)\bar{\nu }\left(dx\right)\right)\right]dt \end{array}$$

$$\begin{array}{cc} \; & \leq E^{Q}\int_{0}^{T}\left[r_{t}^{2}{\tilde{N}}_{t}^{2}+C_{G}^{2}{\left(1+\left|S_{t-}\right|\right)}^{2}+{\overset{ˇ}{r}}_{t}^{2}S_{t-}^{2}+S_{t-}^{4}\int_{R}{\left(e^{y}-1\right)}^{4}\bar{\nu }\left(dx\right)+S_{t-}^{2}\right.\\ \; & \left.\;\;\;\;\;\;+{\tilde{N}}_{t-}^{2}\left(1+S_{t-}^{2}+C_{G}^{2}{\left(1+\left|S_{t-}\right|\right)}^{2}+S_{t-}^{2}\int_{R}{\left(e^{y}-1\right)}^{2}\bar{\nu }\left(dx\right)\right)\right]dt\\ \; & \leq \int_{0}^{T}\left[{\left(E^{Q}r_{t}^{4}\right)}^{\frac{1}{2}}{\left(E^{Q}{\tilde{N}}_{t}^{4}\right)}^{\frac{1}{2}}+{\left(E^{Q}{\overset{ˇ}{r}}_{t}^{4}\right)}^{\frac{1}{2}}{\left(E^{Q}S_{t-}^{4}\right)}^{\frac{1}{2}}+C_{E}E^{Q}S_{t-}^{4}+\left(1+2C_{G}^{2}\right)E^{Q}{\tilde{N}}_{t-}^{2}\right.\\ \; & \left.\;\;\;\;\;\;+\left(1+2C_{G}^{2}+C_{E}\right){\left(E^{Q}{\tilde{N}}_{t-}^{4}\right)}^{\frac{1}{2}}{\left(E^{Q}{\tilde{S}}_{t-}^{4}\right)}^{\frac{1}{2}}+\left(2C_{G}^{2}+1\right)E^{Q}S_{t-}^{2}+2C_{G}^{2}\right]dt\\ \; & \leq T\left(c_{r}^{\frac{1}{2}}n_{4}^{\frac{1}{2}}\right.\left.+c_{\overset{ˇ}{r}}^{\frac{1}{2}}m_{8}^{\frac{1}{4}}b_{8}^{\frac{1}{4}}+C_{E}m_{8}^{\frac{1}{2}}b_{8}^{\frac{1}{2}}+\left(1+2C_{G}^{2}\right)n_{2}+\left(1+2C_{G}^{2}+C_{E}\right)n_{4}^{\frac{1}{2}}m_{8}^{\frac{1}{4}}b_{8}^{\frac{1}{4}}\right.\\ \; & \left.+\left(2C_{G}^{2}+1\right)m_{4}^{\frac{1}{2}}b_{4}^{\frac{1}{2}}+2C_{G}^{2}\right)<\infty . \end{array}$$

Thus, condition (8) is fulfilled. The above equalities and Theorem 2 imply (22). □

## 4. Dupire Formula for Jump-Diffusion and Constant Interest Rate with the Application of the Minimal Entropy Martingale Measure

As previously, we assume that the filtered probability space $\left(\Omega ,F,{\left(F_{t}\right)}_{t\in T},P\right)$ satisfies the usual assumptions.

In this section, we will assume that the instantaneous interest rate is constant. In this case, for some real market parameters, including the distribution of jumps in the price of the underlying asset with respect to the initial probability measure P, it is possible to use the minimal entropy martingale measure as the risk-neutral measure and obtain the Dupire formula for Q of this form. The strengths of using this measure in pricing financial derivatives are presented, inter alia, in [38] (Subsect. 7.4).

Let $S={\left(S\right)}_{t\in T}$ denote a geometric Lévy process of the form

$$S_{t}=S_{0}\exp\left(Y_{t}\right),\;t\in T,$$

modelling the underlying asset price, for a Lévy process ${\left(Y_{t}\right)}_{t\in T}$. Let *r* be a constant instantaneous interest rate and

(35) $$Z_{t}=e^{-rt}S_{t},\;t\in T,$$

represent the discounted price of the underlying asset. In the equivalent martingale measure method (see, e.g., [38]), one has to find a risk-neutral probability measure Q on $\left(\Omega ,F\right)$ equivalent to P such that $Z_{t}$ is a Q-martingale (we will call it an *equivalent martingale measure*). For pricing purposes, one should describe the form of process S with respect to Q. Then, the price process ${\left(c_{t}\right)}_{t\in T}$ of a European option with a pay-off function f is given by

(36) $$c_{t}=\exp\left(-r(T-t)\right)E^{Q}\left(f\left(S_{T}\right)|F_{t}\right).$$

We recall the basic notions and a fact presented in [38] concerning the minimal entropy martingale measure.

We use the symbol $H\left(Q,P\right)$ to denote the relative entropy of Q with respect to P (both defined on $\left(\Omega ,F\right)$) described by the formula

$$H\left(Q,P\right)=\left\{\begin{array}{cc} \int_{\Omega }\log\left[\frac{dQ}{dP}\right]dQ & \;\mathrm{if}\;Q<<P\\ +\infty & \mathrm{otherwise}, \end{array}\right.$$

where the notation Q<<P means that Q is absolutely continuous with respect to P. If for an equivalent martingale measure $P_{0}$, the following inequality holds

$$H\left(P_{0},P\right)\leq H\left(Q,P\right)$$

for an arbitrary equivalent martingale measure Q, then, we call $P_{0}$ the *minimal entropy martingale measure* (MEMM) of S.

From the theory presented in [38] (Subsect. 7.2.1), it follows that if with respect to P,

$$Y_{t}=\mu_{0}t+\sigma dW_{t}^{\left(2\right)}+J_{t},\;t\in T,$$

where σ>0, $W^{\left(2\right)}$ is the standard Brownian motion, and J is a compound Poisson process with an intensity $\lambda_{0}>0$ and the Lévy measure ${\bar{\nu }}_{0}\left(dx\right)=\lambda_{0}\rho_{0}\left(dx\right)$ for a probability distribution $\rho_{0}\left(dx\right)$ on R with $\rho_{0}\left(\left\{0\right\}\right)=0$. Then, the existence of a solution $\gamma^{*}$ of the equation

(37) $$\mu_{0}+\left(\frac{1}{2}+\gamma \right)\sigma^{2}+\lambda_{0}\int_{R}\left(e^{x}-1\right)e^{\gamma (e^{x}-1)}\rho_{0}\left(dx\right)=r$$

implies

(i) The existence of the MEMM $P^{*}$ of S.
(ii) The following form of Y with respect to $P^{*}$
  $$Y_{t}=\left(\mu_{0}+\gamma^{*}\sigma^{2}\right)t+\sigma dW_{t}^{\left(2\right)}+J_{t}^{*},\;t\in T,$$
  where $J^{*}$ is a compound Poisson process with the intensity
  $$\lambda^{*}=\lambda_{0}\int_{R}e^{\gamma^{*}\left(e^{x}-1\right)}\rho_{0}\left(dx\right)$$
  and the Lévy measure ${\bar{\nu }}^{*}\left(dx\right)=\lambda^{*}\rho^{*}\left(dx\right)$ for the probability distribution
  $$\rho^{*}\left(dx\right)=\frac{e^{\gamma^{*}\left(e^{x}-1\right)}\rho_{0}\left(dx\right)}{\int_{R}e^{\gamma^{*}\left(e^{x}-1\right)}\rho_{0}\left(dx\right)}$$
  on R.

Adapting the notation above to the setting in Section 2.4, we have $Q=Q^{t}=P^{*}$, $X=J^{*}$, $\bar{\nu }={\bar{\nu }}^{*}$ and ${\tilde{N}}_{t}=e^{-rt}$, $S_{t}=S_{t}$ for t∈T.

$$S_{t}=S_{0}\exp\left(\int_{0}^{t}\left(\mu_{0}+\gamma^{*}\sigma^{2}\right)du+\int_{0}^{t}\sigma dW_{u}^{\left(2\right)}+\int_{0}^{t}\int_{R}yN\left(du\;dy\right)\right),\;t\in T.$$

By Theorem 1 and (37),

$$\begin{array}{cc} dS_{t} & =S_{t-}\left(rdt+\sigma dW_{t}^{\left(2\right)}+\int_{R}\left(e^{y}-1\right)q\left(dt\;dy\right)\right). \end{array}$$

Therefore, proceeding as in the proof of [14] (Thm. 4.1), we obtain the Dupire formula in $D^{\prime }$ in the considered case, as follows:

(38) $$\begin{array}{c} \begin{array}{cc} \frac{\partial C}{\partial t}= & \frac{{\overset{=}{\sigma }}^{2}\left(t,k\right)k^{2}}{2}\frac{\partial^{2}C}{\partial k^{2}}-rk\frac{\partial C\left(t,k\right)}{\partial k}\\ \; & +\int_{R}e^{y}\left(C\left(t,ke^{-y}\right)-C\left(t,k\right)-\left(e^{-y}-1\right)k\frac{\partial C\left(t,k\right)}{\partial k}\right)\lambda^{*}\rho^{*}\left(dy\right), \end{array} \end{array}$$

i.e., in the form as in [14] (Eqn. (70)) with $\overset{ˇ}{\lambda }\overset{ˇ}{\varphi }\left(dy\right)$ in [14] replaced by $\lambda^{*}\rho^{*}\left(dy\right)$ and, similarly as in the previous section, $\sigma_{t}=\bar{\sigma }\left(t,S_{t-}\right)=\overset{=}{\sigma }\left(t,S_{t-}\right)S_{t-}$, where $\bar{\sigma },\overset{=}{\sigma }:T\times R\to R$ fulfil the assumptions as in Theorem 3.

The same formula was also obtained (without the considerations concerning the application of the MEMM) in [9] as a special case of the Dupire formula for semimartingales.

## 5. Numerical Example

As mentioned earlier, our paper aims to derive the Dupire formula in a mathematically rigorous way under the assumption that the price of the underlying instrument and the spot interest rate are described by the Merton models of 1976 and 1973, respectively. This results in the Dupire equation in the space of distributions. Because of this, numerical computations using the mentioned formula require additional advanced research. However, to present a numerical illustration of the application of the Dupire formula for the Merton jump-diffusion model of an underlying asset with a constant instantaneous interest rate and the effect of replacing this interest rate with its stochastic counterpart, we will recall some results of computations from [20], where a similar case to the one considered in this paper was discussed, although less formally, excluding the verification of certain assumptions. Since the authors did not use the distributional approach, we will write selected formulas from the aforementioned paper as they are presented, preserving the notation used there with some minor changes and corrections.

We will use the symbols $r_{t}$, B(0,t), and $E^{T}$ to denote the risk-free rate available at time *t*, the price of a zero-coupon bond with maturity at time *t*, and the expectation under the forward measure, respectively.

The price process of the underlying asset is given by the following extension of the model considered in [7] to include a stochastic r, as follows:

(39) $$\begin{array}{c} dS_{t}/S_{t-}=\left(r_{t}-q_{t}-\lambda_{t}m_{t}\right)dt+\sigma \left(t,S_{t-}\right)dW_{t}^{S}+\left(J_{t}-1\right)dN_{t}, \end{array}$$

where $q_{t}$ is the dividend rate, $W_{t}^{S}$ is the Brownian motion; $J_{t}$ is a log-normally distributed jump magnitude with the mean $m_{t}+1$ and standard deviation Σ; $N_{t}$ is a Poisson process with intensity $\lambda_{t}$; and $J_{t}$, $N_{t}$, $W_{t}^{S}$, and $r_{t}$ are independent. We will use the symbols $\sigma^{hyb}\left(T,K\right)$ and $\sigma^{det}\left(T,K\right)$ to denote, respectively, the local volatility of the hybrid model, i.e., with stochastic $r_{t}$ for the maturity T and strike K, and its counterpart with deterministic $r_{t}$. Using the Dupire formula [7] (Eqn. (4)), the authors obtained

(40) $$\begin{array}{cc} \; & {\left[\sigma^{det}\left(T,K\right)\right]}^{2}=2\\ \; & \cdot \frac{\partial_{T}C_{T,K}-\left(q_{T}+\lambda_{T}m_{T}-r_{T}\right)K\partial_{K}C_{T,K}-E\left[J_{T}C_{T,K}/J_{T}\right]\lambda_{T}+\left(q_{T}+\lambda_{T}\left(m_{T}+1\right)\right)C_{T,K}}{\partial_{KK}C_{T,K}K^{2}} \end{array}$$

and proved (see [20] (Proposition 2.1)) that

(41) $$\begin{array}{c} {\left[\sigma^{hyb}\left(T,K\right)\right]}^{2}={\left[\sigma^{det}\left(T,K\right)\right]}^{2}-\frac{2B(0,T)}{\partial_{KK}C_{T,K}K^{2}}E^{T}\left[\left(r_{T}^{hyb}-r_{T}^{det}\right)1_{S_{T-}>K}|S_{T-}=K\right], \end{array}$$

where $r_{T}^{hyb}$ and $r_{T}^{det}$ are the stochastic and deterministic interest rates used in (39).

### 5.1. Local Volatility for Constant Interest Rate

The authors of [20] considered the Eurostoxx 50 index. In the beginning, the implied Black–Scholes volatility for this index, presented in Table 1 (see [20] (Table 1)), was obtained, where strikes are presented as percentages of the initial spot.

The implied volatility was then smoothened with application of the two-step parabolic function. For a fixed value of *K*, the best fitting parabola of the form $\sigma_{imp}\left(T,K\right)=a\left(K\right)+b\left(K\right)T+c\left(K\right)T^{2}$. Then, the parabolas in strikes by determining the following values:

$$\begin{array}{cc} \; & a\left(K\right)=\alpha_{1}+\beta_{1}\log\left(\frac{K}{S_{0}}\right)+\gamma_{1}\log{\left(\frac{K}{S_{0}}\right)}^{2}\\ \; & b\left(K\right)=\alpha_{2}+\beta_{2}\log\left(\frac{K}{S_{0}}\right)+\gamma_{2}\log{\left(\frac{K}{S_{0}}\right)}^{2}\\ \; & c\left(K\right)=\alpha_{3}+\beta_{3}\log\left(\frac{K}{S_{0}}\right)+\gamma_{3}\log{\left(\frac{K}{S_{0}}\right)}^{2} \end{array}$$

were obtained, using least square minimization. In the next step, formula (40) was estimated with different parameters as described in [7], and it was applied to compute the local volatility surface given in Table 2 (see [20] (Table 2)). Table 2 shows that “volatility smiles” are especially noticeable for short-term maturities.

### 5.2. Impact of Stochastic Interest Rate on Local Volatility

It is assumed that

(42) $$\begin{array}{c} dr_{t}^{hyb}=\gamma_{t}dW_{t}^{r} \end{array}$$

for a deterministic $\gamma_{t}$, the standard Brownian motion $W_{t}^{r}$, $\langle dW_{t}^{S},dW_{t}^{r}\rangle =\rho_{S,r}dt$, and a constant $|\rho_{S,r}|\leq 1$. To obtain $\gamma_{s}$, the ATM cap volatility was used. The results are presented in Table 3 (see [20] (Table 3)).

In turn, to obtain $\rho_{S,r}$, the authors used the correlation obtained by computation on two years of weekly data between the Eurostoxx 50 index and the 2Y swap rate.

The local volatility for the hybrid model (with stochastic interest rates) was approximated iteratively by a fixed point iteration procedure described in [20] (Sub-Section 2c). Three iterations of this procedure and the following formulas were applied:

$$\begin{array}{cc} \; & \sigma_{loc}^{hyb,1}\left(T,K\right):=\sqrt{{\left[\sigma^{det}\left(T,K\right)\right]}^{2}-2\rho_{S,r}\int_{0}^{T}\sigma^{det}\left(s,K\right)\gamma_{s}ds},\\ \; & \sigma_{loc}^{hyb,2}\left(T,K\right):=\sqrt{{\left[\sigma^{det}\left(T,K\right)\right]}^{2}-2\rho_{S,r}\int_{0}^{T}\sigma_{loc}^{hyb,1}\left(s,K\right)\gamma_{s}ds},\\ \; & \sigma^{hyb}\left(T,K\right)\approx \sigma_{loc}^{hyb,3}\left(T,K\right):=\sqrt{{\left[\sigma^{det}\left(T,K\right)\right]}^{2}-2\rho_{S,r}\int_{0}^{T}\sigma_{loc}^{hyb,2}\left(s,K\right)\gamma_{s}ds}. \end{array}$$

The results are presented in Table 4 (see [20] (Table 6)).

Finally, the impact of the stochastic interest rates is computed by the formula

$$\begin{array}{c} Bias=\sigma^{hyb}\left(T,K\right)-\sigma^{det}\left(T,K\right) \end{array}$$

and the results are presented in Table 5 (see [20] (Table 7)).

From Table 5, it follows that, for the considered data, the bias becomes greater for longer maturities. In particular, the impact is significant (greater than 3%) for 10-year maturity.

## 6. Conclusions

In this paper, we derived and proved the Dupire formula for the European call option in the space of distributions for the underlying asset price described by the jump-diffusion Merton model (1976) and the stochastic instantaneous interest rate. We used a general framework introduced in [14] for the Margrabe option and the generalised Dupire formula proved therein. We also illustrated the possibility of applying the MEMM as the risk-neutral measure in the case of a jump-diffusion model of the underlying asset price and a constant interest rate to obtain the Dupire formula based on market parameters with respect to the physical probability measure P. Future research plans include deriving Dupire equations for other stochastic models of the instantaneous interest rate and other types of options, which is an important issue from both theoretical and practical points of view.

## Figures and Tables

**Table 1:** Implied Black–Scholes volatility.

Expiry \Strike	85%	90%	95%	100%	105%	110%	115%	120%	125%	130%
1M	38.91%	30.34%	26.05%	23.91%	17.84%	17.84%	17.84%	17.84%	17.31%	16.79%
3M	36.56%	29.26%	25.61%	23.73%	18.31%	18.31%	18.31%	18.31%	17.76%	17.23%
6M	32.86%	27.21%	24.39%	23.05%	18.32%	18.32%	18.32%	18.32%	17.77%	17.23%
9M	31.40%	26.47%	24.03%	22.79%	18.45%	18.45%	18.45%	18.45%	17.90%	17.36%
1Y	30.30%	25.98%	23.75%	22.72%	18.72%	18.72%	18.72%	18.72%	18.16%	17.61%
2Y	27.75%	24.71%	23.18%	22.43%	19.01%	19.01%	19.01%	19.01%	18.44%	17.88%
3Y	24.56%	23.31%	22.69%	22.07%	18.94%	18.94%	18.94%	18.94%	18.37%	17.82%
4Y	26.57%	24.60%	23.54%	23.01%	19.97%	19.97%	19.97%	19.97%	19.38%	18.79%
5Y	26.15%	24.53%	23.67%	23.23%	20.34%	20.34%	20.34%	20.34%	19.73%	19.14%
10Y	25.73%	24.46%	23.80%	23.45%	20.71%	20.71%	20.71%	20.71%	20.08%	19.49%

**Table 2:** Local volatility for the deterministic interest rate.

Expiry \Strike	85%	90%	95%	100%	105%	110%	115%	120%	125%	130%
1M	37.94%	29.90%	25.84%	23.78%	17.94%	17.64%	14.49%	14.49%	14.49%	14.49%
3M	32.90%	27.42%	24.63%	23.18%	18.53%	18.46%	18.23%	17.60%	14.25%	14.25%
6M	27.29%	24.27%	22.69%	21.99%	18.30%	18.25%	18.14%	17.93%	16.87%	15.03%
9M	27.34%	24.54%	22.97%	22.17%	18.86%	18.83%	18.74%	18.61%	17.77%	16.72%
1Y	26.19%	24.03%	22.60%	22.17%	19.16%	19.12%	19.05%	18.94%	18.16%	17.30%
2Y	22.08%	22.25%	22.28%	21.83%	19.06%	19.03%	18.97%	18.89%	18.21%	17.47%
3Y	22.55%	22.95%	23.00%	22.70%	20.04%	20.00%	19.95%	19.89%	19.20%	18.49%
4Y	29.46%	26.74%	25.17%	24.98%	22.28%	22.25%	22.20%	22.15%	21.41%	20.66%
5Y	23.71%	23.90%	24.00%	24.01%	21.75%	21.70%	21.66%	21.61%	20.83%	20.16%
10Y	23.13%	23.43%	23.50%	23.35%	20.89%	20.85%	20.81%	20.75%	20.01%	19.33%

**Table 3:** Interest rate volatility.

Time (s)	$\gamma_{s}$
1M	1.04%
3M	1.20%
6M	1.36%
9M	1.26%
1Y	1.17%
2Y	0.99%
3Y	0.90%
4Y	0.85%
5Y	0.81%
10Y	0.81%

**Table 4:** Local volatility for the stochastic interest rate.

Expiry \Strike	85%	90%	95%	100%	105%	110%	115%	120%	125%	130%
1M	37.90%	29.87%	25.80%	23.75%	17.91%	17.60%	14.45%	14.45%	14.45%	14.45%
3M	32.78%	27.31%	24.51%	23.07%	18.41%	18.35%	18.13%	17.49%	14.13%	14.13%
6M	27.01%	24.00%	22.43%	21.74%	18.05%	18.01%	17.90%	17.68%	16.64%	14.79%
9M	26.93%	24.15%	22.58%	21.79%	18.50%	18.46%	18.38%	18.25%	17.43%	16.37%
1Y	25.65%	23.51%	22.09%	21.68%	18.68%	18.64%	18.58%	18.47%	17.71%	16.85%
2Y	21.01%	21.26%	21.34%	20.90%	18.15%	18.12%	18.07%	18.00%	17.33%	16.60%
3Y	21.11%	21.60%	21.70%	21.41%	18.79%	18.75%	18.71%	18.65%	17.97%	17.28%
4Y	28.01%	25.24%	23.63%	23.46%	20.81%	20.77%	20.73%	20.69%	19.96%	19.22%
5Y	21.55%	21.87%	22.04%	22.08%	19.89%	19.85%	19.82%	19.77%	19.00%	18.36%
10Y	19.35%	19.79%	19.93%	19.81%	17.40%	17.36%	17.32%	17.27%	16.55%	15.88%

**Table 5:** Impact of the stochastic interest rate.

Expiry \Strike	85%	90%	95%	100%	105%	110%	115%	120%	125%	130%
1M	0.03%	0.03%	0.03%	0.03%	0.03%	0.03%	0.03%	0.03%	0.03%	0.03%
3M	0.12%	0.12%	0.12%	0.12%	0.11%	0.11%	0.11%	0.11%	0.11%	0.11%
6M	0.28%	0.27%	0.26%	0.26%	0.25%	0.25%	0.24%	0.24%	0.23%	0.24%
9M	0.41%	0.39%	0.38%	0.38%	0.37%	0.37%	0.36%	0.36%	0.35%	0.34%
1Y	0.54%	0.52%	0.51%	0.50%	0.48%	0.48%	0.47%	0.47%	0.46%	0.45%
2Y	1.07%	0.99%	0.94%	0.93%	0.91%	0.91%	0.90%	0.90%	0.88%	0.87%
3Y	1.44%	1.35%	1.30%	1.29%	1.25%	1.25%	1.25%	1.24%	1.23%	1.21%
4Y	1.45%	1.50%	1.54%	1.52%	1.48%	1.48%	1.47%	1.46%	1.45%	1.43%
5Y	2.16%	2.03%	1.96%	1.93%	1.85%	1.85%	1.85%	1.84%	1.83%	1.81%
10Y	3.78%	3.64%	3.57%	3.55%	3.49%	3.49%	3.49%	3.48%	3.46%	3.45%

## Data Availability

No new data were created or analyzed in this study. Data sharing is not applicable to this article.
